# Application of Fault Diagnosis Method Combining Finite Element Method and Transfer Learning for Insufficient Turbine Rotor Fault Samples

**DOI:** 10.3390/e25030414

**Published:** 2023-02-24

**Authors:** Qinglei Zhang, Qunshan He, Jiyun Qin, Jianguo Duan

**Affiliations:** China Institute of FTZ Supply Chain, Shanghai Maritime University, Shanghai 201306, China

**Keywords:** turbine rotor, intelligent fault diagnosis, finite element method, transfer learning, time-frequency diagram

## Abstract

Deep learning has led to significant progress in the fault diagnosis of mechanical systems. These intelligent models often require large amounts of training data to ensure their generalization capabilities. However, the difficulty of obtaining turbine rotor fault data poses a new challenge for intelligent fault diagnosis. In this study, a turbine rotor fault diagnosis method based on the finite element method and transfer learning (FEMATL) is proposed, ensuring that the intelligent model can maintain high diagnostic accuracy in the case of insufficient samples. This method fully exploits the finite element method (FEM) and transfer learning (TL) for small-sample problems. First, FEM is used to generate data samples with fault information, and then the one-dimensional vibration displacement signal is transformed into a two-dimensional time-frequency diagram (TFD) by taking advantage of the deep learning model to recognize the image. Finally, a pre-trained ResNet18 network was used as the input to carry out transfer learning. The feature extraction layer of the network was trained on the ImageNet dataset and a fully connected layer was used to match the specific classification problems. The experimental results show that the method requires only a small amount of training data to achieve high diagnostic accuracy and significantly reduces the training time.

## 1. Introduction

Rotating machines are widely used in mechanical systems and their working environment is complex and changeable. Their operating conditions directly affect the stability of the entire mechanical system; therefore, the fault diagnosis of rotating machines is receiving increasing attention. As a typical rotating machine, a turbine rotor operates under a wide range of variable conditions for a long period of time, which can have serious consequences if it fails.

To gain insight into rotor fault effects, Wang et al. [1] investigated the effects of rotor unbalance and misalignment faults on a two-rotor system. Ebrahimi et al. [2] studied the dynamic responses of unbalanced, flexible rotors. Ren et al. [3] investigated a coupled rotor-bearing system with touching and cracking faults to reveal its dynamic behavior. Zhang et al. [4] studied the dynamic response of the turbine blades. Hong et al. [5] studied the touch response of a single-disk rotor system under additional constraints.

Most rotating machinery fault diagnoses are based on vibration signals, and the processing of faulty mechanical signals using signal-processing techniques is the main method of fault detection. Specifically, signal features are extracted through different signal processing techniques to determine the fault, including improved local mean decomposition (LMD) [6], empirical mode decomposition (EMD) [7], ensemble empirical mode decomposition (EEMD) [8], and variational modal decomposition (VMD) [9]. However, all these feature extraction methods require a priori knowledge, and the feature extraction work directly affects the results of the fault diagnosis. In recent years, the emergence of deep learning theory has reformed intelligent fault diagnosis (IFD) [10]. Since it can automatically extract signal features without prior knowledge, thus freeing human feature extraction, deep learning provides useful tools for processing and analyzing big data [11] and is favored by more and more experts and scholars [12]. Wang et al. [13] proposed an extended deep belief network (EDBN) for fault classification. Zhang [14] and Wu [15] constructed one-dimensional convolutional neural networks to achieve timely and accurate fault detection for bearings and turbine rotors, respectively. Chen et al. [16] combined convolutional neural networks (CNN) with long short-term memory (LSTM) for the fault diagnosis of bearings, and the diagnostic results outperformed other intelligent algorithms based on a priori knowledge.

Although these AI models are powerful tools for troubleshooting mechanical systems, the most important factor for achieving good diagnostic results is that there must be sufficient training samples for the models to learn. The lack of or difficulty in obtaining turbine rotor fault samples poses a new challenge for intelligent fault diagnosis of turbine rotors. Some mechanical equipment failure samples are insufficient and some scholars have conducted research on small or unbalanced data through generative adversarial networks (GANs) and their variants. Liu et al. [17] proposed a diagnostic framework for a GAN and multi-sensor fusion, where generators are used to synthesize data and thus compensate for small samples of category data. The validation was performed on bearing and gearbox datasets and the results showed the good performance of the proposed method. Ding et al. [18] used a GAN to generate synthetic samples related to real samples under small sample conditions and were able to identify different fault types stably, even with fewer training samples, validating the model on bearing and gearbox datasets. Li et al. [19] proposed an ACW-GAN-GP model to address the diagnostic effects of unbalanced datasets. Luo et al. [20] proposed a C-DCGAN model for aiding the generation of synthetic samples for the fault diagnosis of highly unbalanced datasets and validated the model on bearing and gearbox datasets, proving that it exhibits better fault diagnosis capabilities in the case of small samples. Other scholars have expanded datasets using different data enhancement methods [21]. Numerical simulation is another method used to solve the problem of insufficient data samples. Xiang [22] and Liu et al. [23] established finite element models for rotors and bearings, respectively, and expanded the datasets using simulation data.

Transfer learning is another way to solve the small-sample problem. The AI model is trained on the source dataset and only requires a small number of target data samples when migrating to the target dataset, which can cause the model to converge quickly. Chen et al. [24] studied the application of transfer learning on wind turbine fault datasets with unbalanced data and different distributions and achieved good performance. Huang et al. [25] realized the fault diagnosis of rolling bearings using wavelet transform and transfer learning. Li et al. [26] implemented a fault diagnosis of rolling bearings using deep convolutional domain adversarial transfer learning. Zhu et al. [27] proposed a transfer learning method based on multisource domain adaptation and verified it on four bearing datasets, proving the effectiveness of the proposed method.

Because the diagnosis effect of the AI model with insufficient sample data is poor, different scholars have adopted various methods to solve this problem. For large rotating mechanical equipment such as turbine rotors, sample data are lacking and difficult to obtain, which can lead to poor results in the established intelligent diagnostic models.

In this study, the advantages of finite element simulation and transfer learning in small-sample problems are fully utilized to achieve high diagnostic accuracy for turbine rotors with insufficient fault data, and the proposed FEMATL method can effectively solve the problem of insufficient samples.

The specific arrangement of this paper is as follows. Section 2 introduces the basic theory and fundamental ideas of the FEMATL method and Section 3 introduces the experimental bench equipment for the rotor. The results of the FEMATL method for a specific case are presented in Section 4. The conclusions of this study are presented in Section 5.

## 2. Introduction to the FEMATL Method

Aiming at the problem of the difficulty in training AI models due to insufficient turbine rotor samples, the FEMATL method proposed in this study can effectively solve the problem of insufficient turbine rotor fault samples, accelerate the model training speed, and improve the diagnosis accuracy. This section describes the techniques used in this method and specifies the details of the FEMATL method.

### 2.1. Rotor Failure Mechanism

The rotor-bearing system is formed by a combination of a rotor shaft, disc, and plain bearing. The support at both ends of the spindle is provided by plain bearings and the disc is located at the center of the shaft and connected to the shaft using an interference fit.

By integrating the rotating shaft, disc, and bearings, the dynamic equation of the system can be expressed as:

(1) $$M\ddot{X}+(C+\omega G)\dot{X}+KX=f$$

where *M* is the mass matrix, *C* is the damping matrix, *G* is the gyroscopic matrix resulting from rotor rotation, *K* is the stiffness matrix of the rotor, and *f* denotes the failure force, including the unbalanced force of the rotor and the force resulting from the misalignment failure. *X* is the displacement matrix and *ω* is the rotational speed.

Common failures of rotors include rotor misalignment, rubbing, and rotor unbalance. According to three common fault mechanisms [28,29], when the rotor misalignment fault is considered, the misalignment force acts on the rotating shaft through coupling and the generated excitation forces *f_c_*(*x*) and *f_c_*(*y*) are simultaneously applied to the rightmost node of the rotor. *f_c_*(*x*) and *f_c_*(*y*) can be obtained using the following equations:

(2) $$\left\{\begin{array}{c} f_{c}(x)=m_{c}\cdot c\cdot \omega^{2}\cdot \cos(2\cdot \omega \cdot t)\\ f_{c}(y)=m_{c}\cdot c\cdot \omega^{2}\cdot \sin(2\cdot \omega \cdot t) \end{array}\right.$$

where *m_c_* is the coupling mass, *c* represents the parallel offset distance between the two halves of the rotor, and *ω* represents the rotational speed of the rotor.

A rotor rubbing fault generally occurs in a rotor-bearing system with radial clearance, specifically manifested as the contact and separation between the rotor and stator of rotation. In the rotation process, when a rub-impact fault occurs between the rotor and stator, an instantaneous impact is generated. According to [29], the impact force can be equivalent to a triangle and acts on the rotating shaft.

When an unbalance failure occurs in the rotor, the centrifugal forces *f_m_*(*x*) and *f_m_*(*y*) generated by the unbalance of the rotor are applied in the X and Y directions of the disk mass unit Mass21, respectively. The unbalanced force is

(3) $$\left\{\begin{array}{c} f_{m}(x)=m_{\delta }\cdot \delta \cdot \omega^{2}\cdot \cos(\omega \cdot t+\varphi_{u})\\ f_{m}(y)=m_{{}_{\delta }}\cdot \delta \cdot \omega^{2}\cdot \sin(\omega \cdot t+\varphi_{u}) \end{array}\right.$$

where *δ* denotes the eccentric distance, *ω* denotes the rotational speed, *φ_u_* denotes the initial phase angle, and *m_δ_* denotes the eccentric mass.

### 2.2. Time-Frequency Analysis Method

To transform the one-dimensional vibration signal of the turbine rotor-bearing system, which is not in an image format, into data in an image format, four time-frequency analysis methods were considered in this study: the short-time Fourier transform (STFT) [30], continuous wavelet transform (CWT) [31], generalized S-transform (GST) [32], and Wigner–Ville distribution (WVD) [33].

STFT:

When the signal passes through the STFT, a spectrum diagram is generated and the STFT can map the signal to a two-dimensional function on the time-frequency plane:

(4) $$H(t,f)=\int_{-\infty }^{\infty }x(\tau )g(\tau -t)e^{-j2\pi f\tau }d\tau$$

where *x*(*τ*) is the time signal being analyzed, *t* is the moment of analysis, *τ* is the time constant, *f* is the frequency, *g*(*t*) is the window function, and the mode square of the coefficient *H* of the STFT is called the spectrogram.

CWT:

When the signal passes through the CWT, a wavelet time-frequency diagram is generated. The CWT uses a wavelet basis function to map the signal to a 2D function in the plane where the scale factor and translation factor are located.

(5) $$Z(a,b)=\frac{1}{\sqrt{b}}\int_{-\infty }^{\infty }x(t)h(\frac{t-a}{b})dt$$

where *a* is the translation factor, *b* is the scale factor, *h*(*t*) is the wavelet basis function, and the mode square of the coefficients *Z* of the CWT is called the wavelet time-frequency diagram.

GST:

Based on the S-transform, the GST improves the time-frequency resolution of the S-transform by introducing the parameters *λ* and *p* to adjust the time continuation degree and attenuation trend of the window function. The formula is as follows:

(6) $$S(\tau ,f)=\int_{-\infty }^{\infty }x(t)\frac{\lambda |f{|}^{p}}{\sqrt{2\pi }}e^{\left(-\frac{\lambda^{2}f^{2p}{\left(\tau -t\right)}^{2}}{2}\right)}e^{\left(-i2\pi f\right)}dt$$

where *λ* > 0 and *p* > 0. When *λ* = 1 and *p* = 1, this is the standard S-transform. *x*(*t*) is the time signal being analyzed, *f* is the frequency, and *τ* is the translation.

WVD:

The WVD is a quadratic time-frequency distribution and the WVD of *x*(*t*) of the signal is defined as

(7) $$W(t,f)=\int_{-\infty }^{\infty }x(t+\frac{\tau }{2})x^{*}(t-\frac{\tau }{2})e^{-j2\pi f\tau }d\tau$$

where *x*(*t*) is the time signal and * denotes the complex conjugate. Because *x*(*t*) appears twice, it is called a bilinear transformation. The equation does not contain any window functions, thus avoiding the contradiction that its time and frequency resolutions cannot be reconciled.

### 2.3. Deep Residual Network (ResNet)

**Figure 1:**
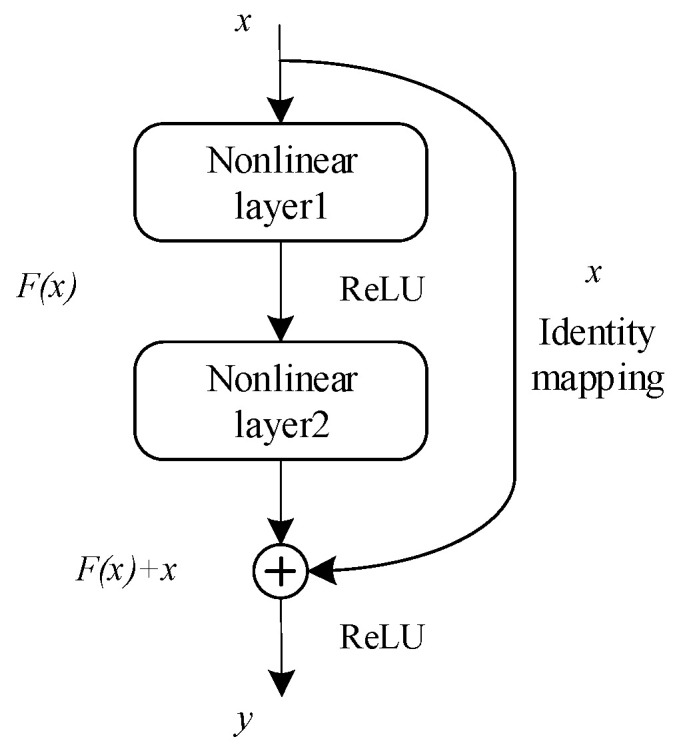
Diagram of residual blocks.

To address the difficulty of training neural networks as the depth of a model increases, He et al. [34] proposed the Residual Network (ResNet). The ResNet network is composed of multiple residual blocks stacked on top of each other. The residual blocks are shown in Figure 1.

Through the residual connection, the input x of the neural network is directly connected to the output of the nonlinear layer through an identity map. Thus, the residual module only needs to learn the parts that are different between the input and output, simplifying the network learning task and improving the discrimination ability of the network. The learning process is as follows:

(8) y=F(x)+x

Owing to the residual connectivity, the ResNet network significantly alleviates the problems associated with deeper network layers, allowing deeper networks to be built and improving the stability of model training.

**Figure 2:**
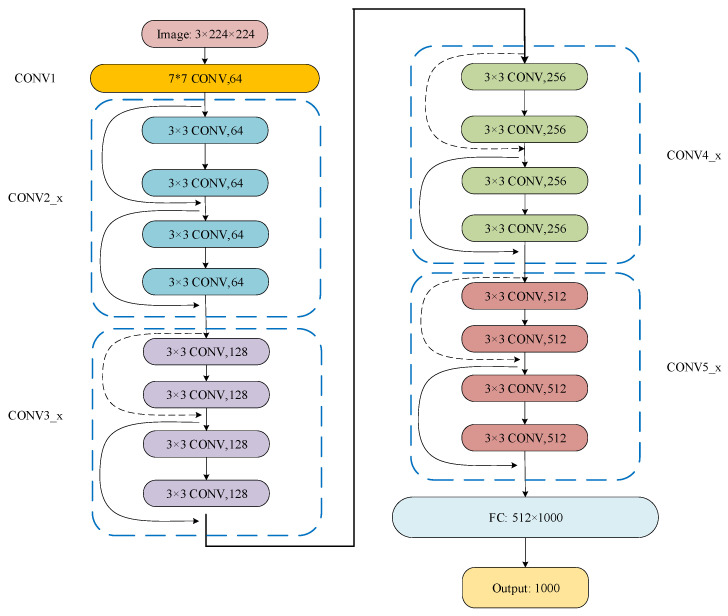
ResNet18 network structure diagram.

In this paper, the ResNet18 [35] network is used for model training. The basic architecture of ResNet18 is a ResNet and the depth of the network is 18 layers so it is called ResNet18. The network structure is shown in Figure 2. It consists of 17 convolutional layers and one fully connected layer. The “3 × 3 CONV, 64” indicates that the convolutional kernel size of the convolutional layer is 3 × 3 and the number of convolutional kernels is 64.

### 2.4. Transfer Learning and ImageNet Dataset

#### 2.4.1. Transfer Learning

Transfer learning is the knowledge learned from data in the source domain to improve or optimize the learning effect of the target prediction function in the target domain [36]. The correlation between data from two different domains is exploited to transfer knowledge from the source domain to the target domain, thereby reducing the training time or improving the accuracy.

**Figure 3:**
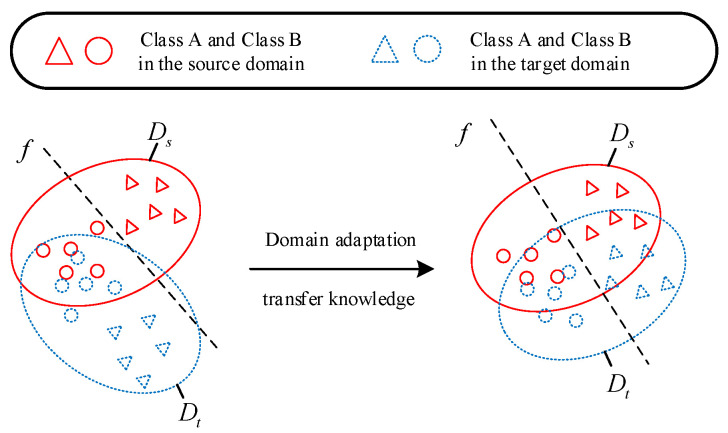
Schematic diagram of transfer learning.

Given a source domain *D_s_* = {*X_s_, P_s_*(*X*)} and task *T_s_* = {*Y_s_, f_s_*(*·*)} and a target domain *D_t_* = {*X_t_, P_t_*(*X*)} and task *T_t_* = {*Y_t_, f_t_*(*·*)}, the purpose of transfer learning is to help or improve the prediction function *f*(*·*) in the target domain with the knowledge learned in the source domain. Generally, it is assumed that the source and target domains are not identical. A schematic of the transfer learning between the source and target domains is shown in Figure 3.

#### 2.4.2. ImageNet Dataset

Instead of training network models from scratch, many people working in the field of computer vision often use open source models pre-trained on the ImageNet dataset [37] and migrate them to local models using transfer learning to shorten the model training time and achieve better results. There are 1000 categories in the ImageNet dataset, including animals, plants, buildings, vehicles, and more. There are more than 1000 images in each category of which about 1.2 million images are used as the training set, more than 50,000 images are used as the validation set, and more than 100,000 images are used as the test set. The ImageNet dataset is rich in categories and has a huge number of images. A network trained on the ImageNet dataset can extract general features from images, which can improve the recognition ability of the network. In this paper, the ResNet18 network is pre-trained on the ImageNet dataset and then migrated to the turbine rotor fault dataset for fine-tuning, which significantly shortened the training time and improved the network’s diagnostic ability.

### 2.5. The Proposed Method

**Figure 4:**
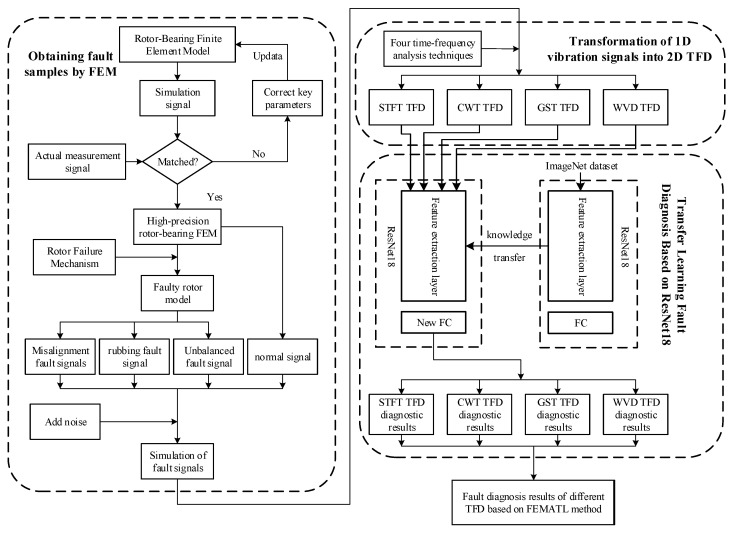
Flow chart of turbine rotor fault diagnosis method based on FEMATL method.

Figure 4 shows a flow diagram of the FEMATL method. This method can effectively solve the problem of insufficient turbine rotor fault samples, accelerate the model training speed, and improve diagnostic accuracy. The method is roughly divided into three steps: finite element simulation to obtain fault samples, transformation of 1D vibration signals into 2D time-frequency maps, and transfer learning fault diagnosis based on ResNet18. The technical details are described in detail below.

Step 1: Finite element simulation to obtain fault samples.

(1) Construct a finite element model of the turbine rotor-bearing system. The finite element software ANSYS was used to build the model and generate the simulated vibration signals. The beam element was used to simulate the rotating shaft, the mass element to simulate the disc, and the bearing element to simulate the sliding bearings at both ends of the rotating shaft.

(2) Modify the rotor-bearing finite element model. Since a perfectly balanced rotor cannot exist, the eccentric load caused by the rotor eccentricity acts on the rotor and causes it to vibrate. In order to ensure that the finite element model matched the actual rotor model, it was necessary to set a comprehensive eccentricity for the rotor and continuously correct this eccentricity so that the finite element simulation signal and the actual rotor measurement signal matched. In order to measure the matching degree between the analog signal and the measured signal, cosine similarity was used to measure the similarity between them. Cosine similarity maps two curves to a vector space and calculates the cosine value of the angle between two vectors. The closer the cosine value is to 1, the more similar the two curves are. The cosine similarity calculation formula of two vectors *x* and *y* is:

(9) $$\cos\langle x,y\rangle =\frac{\sum_{i=1}^{n}x_{i}y_{i}}{\sqrt{\sum_{i=1}^{n}x_{i}^{2}}\cdot \sqrt{\sum_{i=1}^{n}y_{i}^{2}}}$$

where *x_i_* and *y_i_* are the *i*-th elements of vectors *x* and *y*, respectively. After this step, the finite element model of the rotor-bearing system was built to reflect the actual rotor vibration.

(3) Simulation to generate the fault samples. According to the rotor fault mechanism, the corresponding excitation force was applied to the rotor-bearing finite element model to simulate and generate different types of fault signals. To simulate the vibrations of actual machinery and equipment, Gaussian noise with a certain signal-to-noise ratio was artificially added to the vibration signal.

Step 2: Transformation of a one-dimensional vibration signal into a two-dimensional time-frequency diagram.

To take advantage of deep learning in image processing, the 1D vibration signal generated by the simulation was transformed into a time-frequency diagram in RGB format. In order to study the influence of different time-frequency analysis techniques on rotor fault diagnosis, the one-dimensional vibration signals were generated as short-time Fourier transform time-frequency diagrams (STFT TFD), continuous wavelet transform time-frequency diagrams (CWT TFD), generalized S-transform time-frequency diagrams (GST TFD), and Wigner–Ville distribution time-frequency diagrams (WVD TFD) according to the different time-frequency analysis methods.

Step 3: Transfer learning fault diagnosis based on ResNet18.

The ResNet18 network was pre-trained on the ImageNet dataset and the feature extraction part of the network had shallow features for extracting the general images. In order to transfer the knowledge learned by the network on ImageNet, in this experiment, the weight parameters of all layers of the network, except for the fully connected layer, were frozen and the fully connected layer was modified to match the brand-new fully connected layer in this experiment. The input of the ResNet18 network, which was the time-frequency map data obtained in step 2, was used and the whole model was retrained.

## 3. Introduction of Rotor Test Bench

In this section, the rotor experiment platform is introduced to provide a physical basis for the subsequent construction of the finite element model of the rotor-bearing system and the key parameters of the finite element model are modified through the measured signals of the rotor experiment platform.

**Figure 5:**
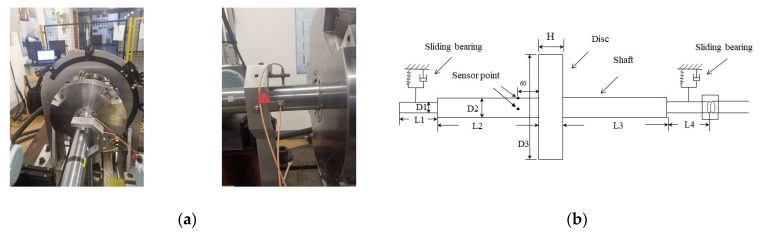
Rotor-bearing system experimental platform: (**a**) Experimental platform. (**b**) Dimensions of the rotor model, where L1 = 140 mm, L2 = L3 = 435 mm, L4 = 180 mm, H = 50 mm, D1 = 50 mm, D2 = 60 mm, D3 = 350 mm, and the two displacement sensors are located 60 mm to the left of the turntable.

Due to the complex structure and large structure size of actual turbine rotors, limited by experimental conditions and considerations of test safety, the incomplete geometric similarity of turbine rotors is often used to reduce its geometric size, thus simplifying the structure of prototype rotors and making the model easier to verify [38]. The similarity theory was used to realize the relationship between the prototype system and the simplified model. In recent years, there has been increasing interest among experts and scholars in rotor dynamics research based on similarity theory [39,40,41]. By using similarity theory to design and manufacture a model similar to the prototype, the physical characteristics of the prototype can be reproduced through model experiments and the physical nature and law of the prototype can be revealed [42]. The rotor used in this experiment was a simplified model of the turbine rotor. In the simplification process, we focused mainly on matching the first- and second-order natural frequencies of the experimental rotor to those of the prototype turbine rotor. Through CAE analysis, the first two natural frequencies of the model rotor were the same as those of the turbine rotor. The established model rotor is shown in Figure 5a. The rotor-bearing system is composed of a rotary table, a rotating shaft, and sliding bearings at both ends. The servo motor provides the rotating power of the rotor and lubricating oil is injected into the sliding bearing through the hydraulic system to provide oil-film support during rotor rotation. Two eddy current displacement sensors were placed in the X- and Y-directions of the rotating axis, respectively, at a distance of 60 mm from the disc in a 90° symmetry to collect the vibration signals during the rotation. Figure 5b shows the dimensional data of the rotor. The sampling frequency of the sensor was set to 2000 Hz and the motor rotation speed was 1000 rpm.

The vibration signal measured by the rotor experimental platform was used as a reference for the simulation signal of the finite element model. By constantly modifying the finite element model, the simulation signal gradually approximates the measured signal. Finally, a finite element model of the rotor bearing with high precision was obtained.

## 4. Results and Discussion

This section verifies the effectiveness of the FEMATL method for a specific case. First, a finite element model of the rotor-bearing system is constructed according to the rotor experimental platform and data samples with fault information are generated based on this model. Then, the vibration signal is transformed into four different time-frequency diagrams. Finally, the ResNet18 network pre-trained on ImageNet was used for transfer learning fault diagnosis. The specific experimental contents are detailed below.

### 4.1. Finite Element Simulation to Obtain Fault Samples

#### 4.1.1. Finite Element Model of Rotor-Bearing System

In this study, the commercial finite element software ANSYS was used to establish the finite element model of the rotor-bearing system, and the material parameters of the rotor are listed in Table 1. The rotary shaft was simulated using the Beam189 element, the rotary table was simulated using the Mass21 element, and the Combi214 element was simulated using a sliding bearing. The unit types are listed in Table 2.

**Figure 6:**
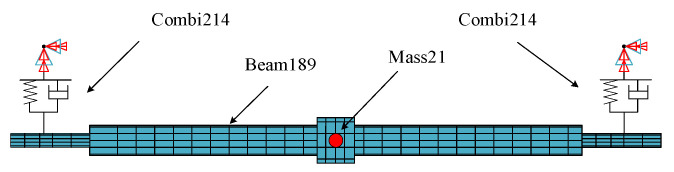
Finite element model of rotor bearing system.

The finite element model of the rotor-bearing system established according to the above parameters is shown in Figure 6.

#### 4.1.2. Modifying the Finite Element Model of the Rotor-Bearing System

Based on the vibration signals generated by the established finite element model of the rotor-bearing system, the vibration signals were compared with the measured signals of the experimental platform of the rotor-bearing system and the key parameters of the model were modified to match the measured signals.

As a rotor experiences wear owing to machining errors and long-term operating conditions, the rotor has a certain amount of eccentricity and the eccentric load acts on the whole rotor, which causes the rotor to vibrate. To accurately obtain the eccentric load, the eccentric distance *e* of the rotor must be known. To obtain the rotor eccentricity under normal conditions, the eccentric load (i.e., the unbalanced force) can be applied to the finite element model of the rotor bearing, the finite element simulation signal is compared with the measured signal of the actual rotor, and the eccentricity is constantly updated.

The eccentric force is decomposed in the X- and Y-directions according to Equation (3) and acts on the disc (Mass21) simultaneously. where m_δ_ = 59.71 kg is the total mass of the rotor, δ is the integrated eccentricity, ω = 104.72 rad/s is the rotational speed of the rotor, and the initial phase *φ_u_* = 0.

**Figure 7:**
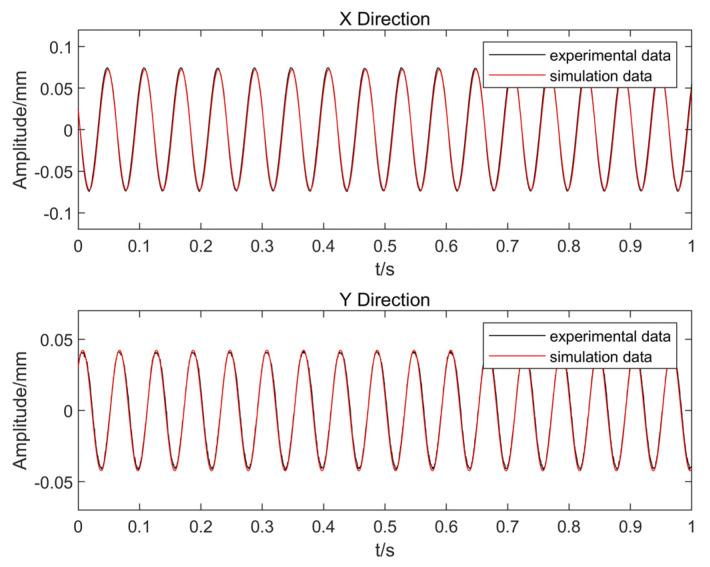
Comparison of experimental and simulated signals in the X- and Y-directions.

The boundary conditions of the finite element model were set to be consistent with those of the experimental rotor and the vibration signals of the simulation model were set to be consistent with those of the experimental rotor by continuously modifying the comprehensive eccentricity of the rotor. Figure 7 shows the experimental vibration signal and simulated vibration signal when the integrated eccentricity was 0.4 mm.

At this point, the cosine similarities of the simulated and measured signals in the X- and Y-directions were 0.999 and 0.998, respectively. The finite element model of the rotor-bearing system can be revised using the above modifications so that the model can correctly reflect the actual running status of the rotor; therefore, the revised integrated eccentricity was 0.4 mm.

#### 4.1.3. Obtaining Fault Samples

The finite element model of the rotor-bearing system obtained using the above method can effectively reflect the vibration of a real rotor. Based on the rotor failure mechanism, the corresponding excitation force is applied to the modified rotor bearing finite element model, and different types of failure samples can be obtained. In this study, three fault states of the turbine rotor system were considered, including the rotor misalignment, rub impact, and rotor unbalance, with each state having three different severities, resulting in a total of nine fault types. This study also considered the health type without failure. The specific method is described below.

(1) Misalignment

In this experiment, only the parallel misalignment of the rotor was considered. According to Equation (2), the excitation forces *f_c_*(*x*) and *f_c_*(*y*) generated by the misalignment were applied to the rightmost node of the rotor simultaneously. The mass of the coupling was set at *m_c_* = 1.8 kg; the parallel offset distances *c* were set at 1, 1.5, and 2 mm; and the speed ω = 104.72 rad/s.

(2) Rubbing

**Figure 8:**
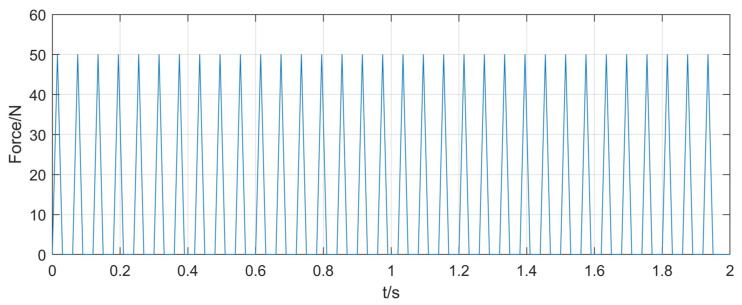
50 N rubbing force in X- and Y-directions.

According to the rotor rub-impact fault theory and the method in [29], the impact force generated by the rub impact between the rotor and stator is equivalent to a triangular pulse wave. In this case, impulse forces of 50 N, 70 N, and 90 N were applied simultaneously in the X- and Y-directions to simulate three types of rubbing-fault severities. Figure 8 shows the rubbing forces in the X- and Y-directions for the first degree of the rubbing fault.

(3) Unbalance

According to Equation (3), the centrifugal forces *f_m_*(*x*) and *f_m_*(*y*) generated by rotor unbalance are applied in the X- and Y-directions of mass element Mass21, respectively. In the formula, the eccentricity δ = 0.1 m, the rotational speed ω = 104.72 rad/s, the initial phase *φ_u_* = 0, and the eccentricity masses *m_δ_*= 0.1, 0.2, and 0.3 kg.

**Figure 9:**
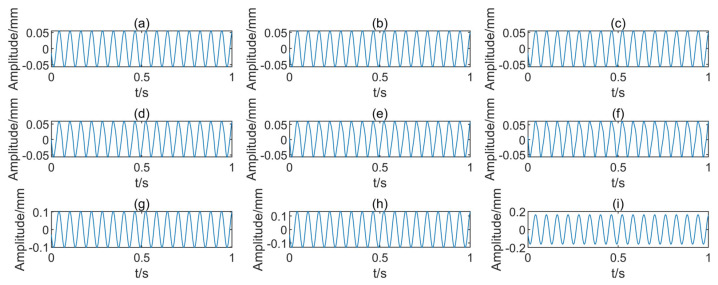
Nine faulty rotor simulation signals: (**a**–**c**) three misalignment faults, (**d**–**f**) three rubbing faults, (**g**–**i**) three unbalance faults.

For the modified finite element model of the rotor-bearing system, the excitation forces caused by the above nine faults were applied to the model and the displacement data in the X-direction were selected as the fault samples. Table 3 lists the nine fault types and one health type along with the corresponding labels. Figure 9 shows the simulation signals of the nine types of faulty rotors.

**Figure 10:**
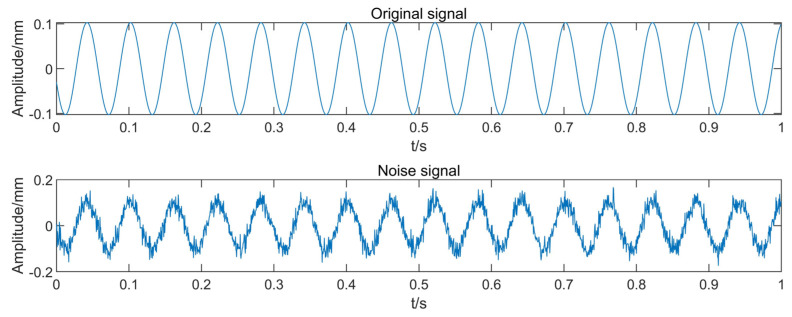
Original signal and noisy signal with SNR = 10.

In practical applications, mechanical equipment inevitably produces a certain amount of noise. This noise may interfere with the environment and machinery itself. To reflect the effect of noise more accurately, Gaussian white noise with a signal-to-noise ratio of 10 was artificially added to the simulated signal. The signal-to-noise ratio SNR is defined as follows:

(10) $$SNR=10\log_{10}(\frac{P_{s}}{P_{n}})$$

where *P_s_* denotes the signal power and *P_n_* denotes the power of the Gaussian noise. The signal with added noise and the original signal are shown in Figure 10 (with the rotor unbalance fault signal as the example).

Fault samples were created for each fault signal with a sample length of 2048 data points. Each fault type consisted of 500 samples and a total of 5000 samples were used. The training, validation, and test sets were created using a ratio of 3:1:1. The training set consisted of 3000 samples, and the verification set and test set each consisted of 1000 samples.

### 4.2. Transformation of One-Dimensional Vibration Signal into Two-Dimensional Time-Frequency Diagram

**Figure 11:**
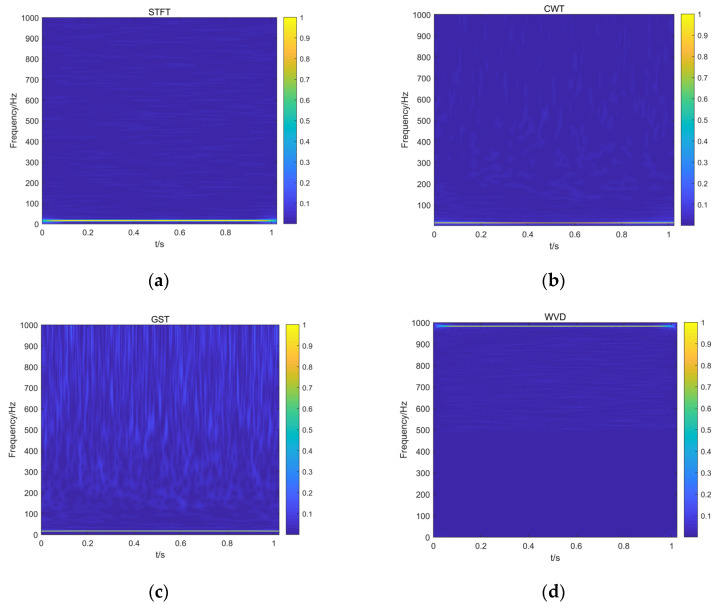
Time-frequency diagrams transformed by four different time-frequency analysis methods. (**a**) STFT time-frequency diagram, (**b**) CWT time-frequency diagram, (**c**) GST time-frequency diagram, (**d**) WVD time-frequency diagram.

The vibration signals generated by the finite element model of the rotor-bearing system were one-dimensional data that could not be directly used as the input of the ResNet18 network. To take advantage of the deep learning model for image processing, four time-frequency analysis techniques (STFT, CWT, GST, and WVD) were used to transform the one-dimensional vibration displacement signal into a time-frequency diagram. Figure 11 shows four different time-frequency diagrams of the same signal.

The fault signals of each type were converted into different time-frequency graphs using the above methods and four different datasets were generated, including the STFT TFD dataset, CWT TFD dataset, GST TFD dataset, and WVD TFD dataset. Each dataset contained sample pictures of 10 fault types and the dimensions of each picture were 875 × 656 × 3. There were 3000 pictures in the training set, 1000 pictures in the verification set, and 1000 pictures in the test set. Table 4 describes the different rotor fault datasets in detail.

### 4.3. Turbine Rotor Fault Diagnosis Based on ResNet18

#### 4.3.1. Intelligent Diagnosis of Fault Data Based on Transfer Learning with ResNet18

**Figure 12:**
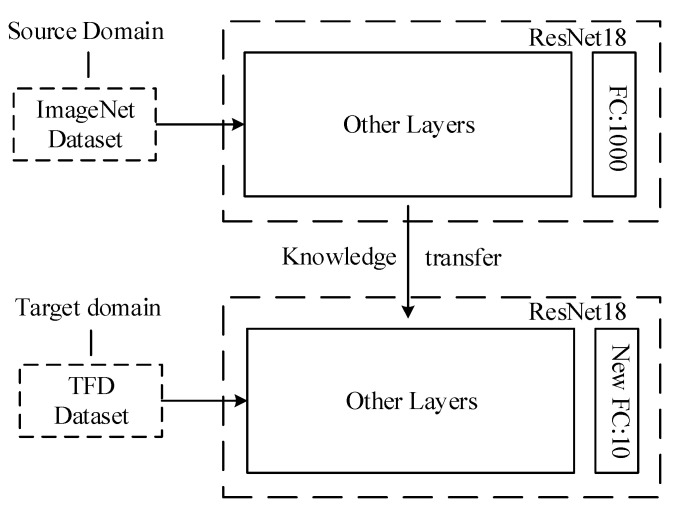
Diagram of transfer learning from source domain to target domain.

The ResNet18 network was pre-trained on the ImageNet dataset and the feature extraction part of the network extracted the shallow features of each general image. Transferring this capability to the time-frequency graph dataset accelerated the training speed of the network in the target domain and improved training accuracy. To successfully transfer the network from the source domain to the target domain, the fully connected layer of the network was processed by replacing the fully connected layer of the network on the source domain with a new fully connected layer that matched the target domain classification task, as shown in Figure 12.

The specific operation was to freeze the weight parameters of the network trained on the ImageNet data and replace the original fully connected layer of 1000 neurons with a fully connected layer of 10 neurons. After the pre-training weights were loaded, the time-frequency diagram dataset was input into the network and the model was trained from beginning to end.

**Figure 13:**
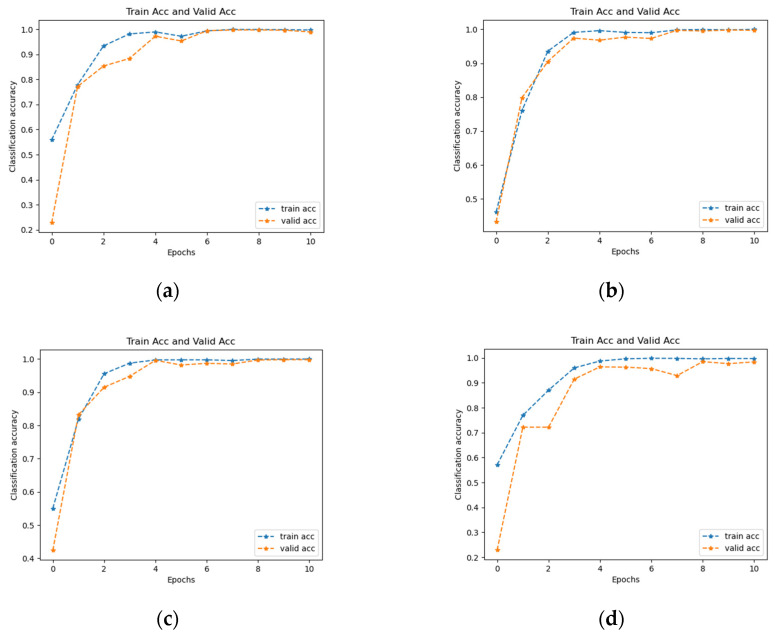
Accuracy curves for different time-frequency diagram datasets: (**a**) STFT time-frequency diagram dataset, (**b**) CWT time-frequency diagram dataset, (**c**) GST time-frequency diagram dataset, (**d**) WVD time-frequency diagram dataset.

In this training, the batch size of each training was set at 128. The Adam [43] optimization algorithm was adopted and the learning rate was set at 0.0001. The cross-entropy loss function was used and the epochs were set to 10. Figure 13 shows the accuracy curves of the training process for each of the four time-frequency diagram datasets. “Acc” in the following images refers to the accuracy.

**Figure 14:**
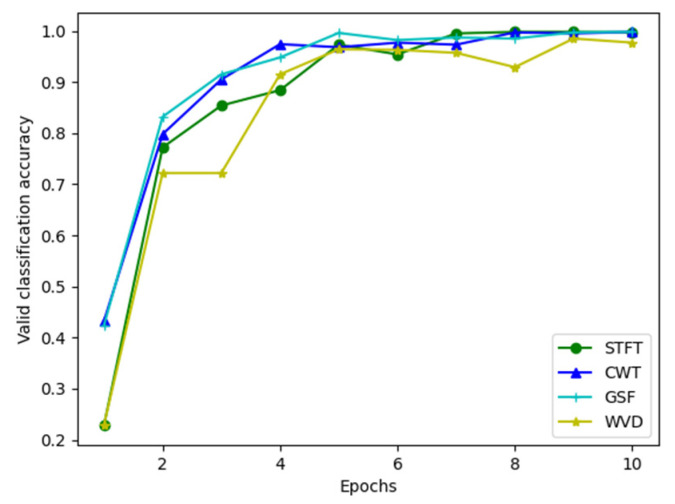
Accuracy of the validation set during the iterations of different time-frequency diagrams.

Table 5 shows the accuracy of the validation set for each iteration. Figure 14 shows the accuracy curves of the validation set during the iterations of the different time-frequency diagram datasets. By combining Table 5 and Figure 14, it can be seen that when the number of iterations was 10, the accuracy of the validation set of the STFT, CWT, and GST TFDs all reached 99.8%, whereas the WVD TFD dataset performed slightly worse, reaching 98.5%. For the four datasets, the ResNet18 network considerably shortened the training time, accelerated the convergence of the model, and achieved high accuracy in a very small number of iterations, indicating that the network successfully transferred knowledge from the ImageNet dataset to the experimental dataset.

**Figure 15:**
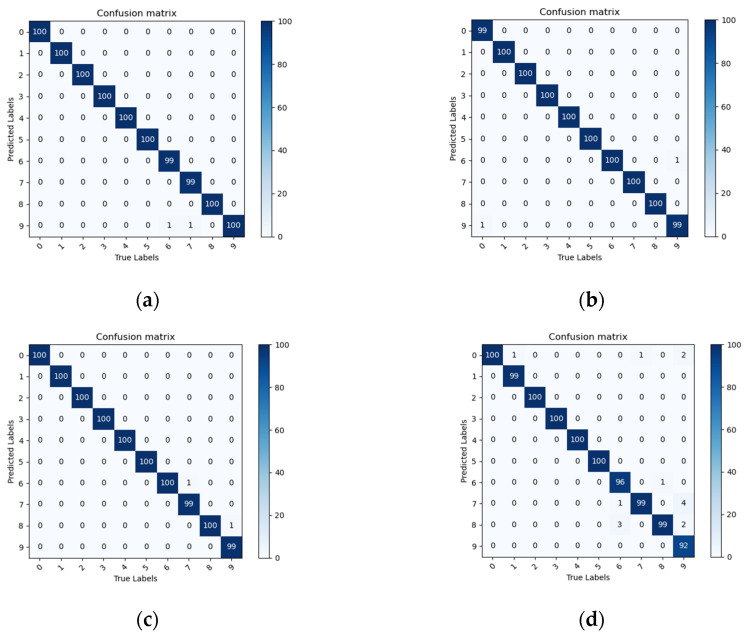
Multi-classification confusion matrix for transfer learning based on ResNet18 network. (**a**) STFT confusion matrix, (**b**) CWT confusion matrix, (**c**) GST confusion matrix, (**d**) WVD confusion matrix.

In this study, a multi-classification confusion matrix was also introduced to compare and analyze the transfer learning fault diagnosis based on ResNet18, as shown in Figure 15. The values on the main diagonal represent the number of correctly identified samples and the other values represent the number of incorrectly identified samples.

As can be seen in Figure 15, for the four different TFD datasets, only individual samples of the ResNet18 network were identified incorrectly, whereas the rest of the samples were identified correctly, which further indicates that the transfer learning fault diagnosis can effectively identify the fault types.

Precision, recall, specificity, and F1 value are common evaluation indexes for deep learning fault diagnosis by which the effectiveness of the diagnosis can be further evaluated. The formulas for the different evaluation indexes are:

(11) $$\left\{\begin{array}{l} Precision=\frac{TP}{TP+FP}\\ Recall=\frac{TP}{TP+TN}\\ Specificity=\frac{TN}{TN+FP}\\ F1=\frac{2\times Precision\times Recall}{Precision+Recall} \end{array}\right.$$

In the formulas, *TP* represents a true positive, *TN* represents a true negative, *FP* represents a false positive, and *FN* represents a false negative. The evaluation indexes of the CWT TFD dataset are shown in Table 6. It can be seen from the table that the evaluation index of each fault classification is above 0.99, which fully indicates that the network has excellent diagnostic performance for the rotor fault dataset, and proves the effectiveness of the proposed method.

**Figure 16:**
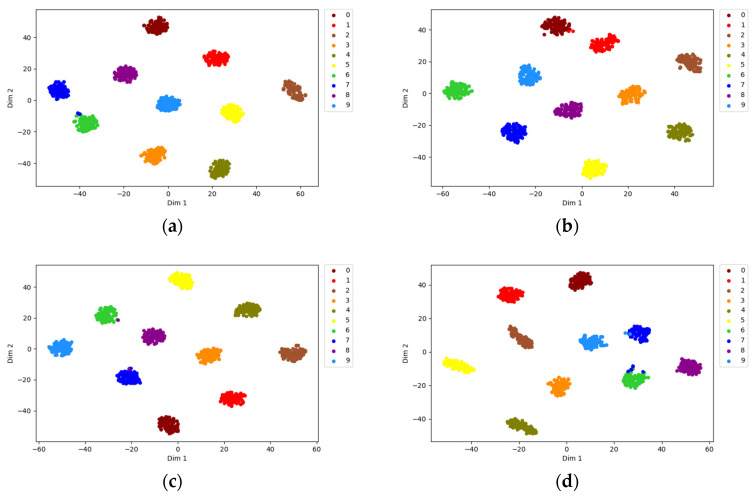
t-SNE visualization of the fully connected layer of the ResNet18 network. (**a**) STFT dataset, (**b**) CWT dataset, (**c**) GST dataset, (**d**) WVD dataset.

To illustrate the powerful recognition ability of the migration learning-based ResNet18 network for different classes of time-frequency graphs, the fully connected layer of ResNet18 was visualized by t-SNE dimensionality reduction, as shown in Figure 16. The t-SNE is a dimensionality reduction technology that can reduce high-dimensional data to low-dimensional data. It is generally used for data visualization. t-SNE diagrams can more intuitively verify the effectiveness of the algorithm.

As shown in Figure 16, after the sample was trained on the ResNet18 network, the feature data points of the same fault type were clustered together, which differs from other fault types. This proves that the transfer learning fault diagnosis network based on ResNet18 can effectively identify different types of faults.

#### 4.3.2. Intelligent Diagnosis of Fault Data Using Resnet18 without Transfer Learning

**Figure 17:**
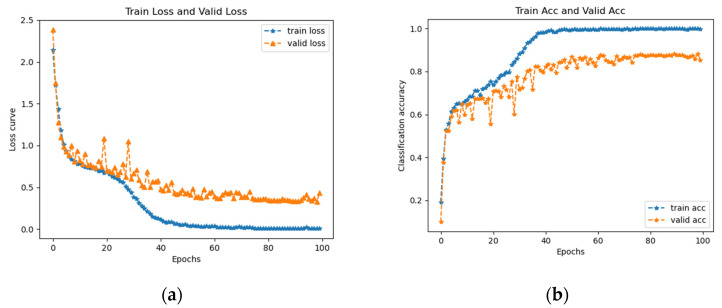
Loss and accuracy curves of ResNet18 network without migration learning for CWT time-frequency diagrams. (**a**) Loss curve. (**b**) Accuracy curve.

To illustrate the advantages of transfer learning in this experiment, a fault diagnosis of the ResNet18 network without using a pretrained model was also performed in this study. After randomly initializing all the weight parameters of the ResNet18 network, one of the time-frequency diagram datasets was used as the target data input to the network for training, and the loss versus accuracy curve of its training is shown in Figure 17. The selected time-frequency diagram data were CWT time-frequency diagrams.

When the iteration was 100 times, the accuracy of the verification set reached a maximum of 0.882, which was not as high as the 10 iterations of the ResNet18 network based on transfer learning. Table 7 summarizes the impact of the four TFD datasets with and without transfer learning. It can be seen in the table that convergence of the network was accomplished with a very small number of iterations using transfer learning and the accuracy was higher than the diagnostic results without transfer learning. The training time of the model based on transfer learning was much less than that without transfer learning. Since the fault diagnosis based on transfer learning has higher diagnostic accuracy and less training time, it can meet the requirements of practical engineering.

## 5. Conclusions

To address the problem of insufficient samples in the fault detection of turbine rotor-bearing systems that affect the training effectiveness of AI models, this study proposes a fault detection method for turbine rotors based on FEMATL. First, a finite element model of the rotor-bearing system was constructed using the finite element method and the model was modified based on the actual rotor measurement signal. For the modified model, different types of fault signals were generated through simulations according to the relevant fault mechanism. Then, to give full play to the image recognition advantages of the deep learning model, the generated one-dimensional vibration displacement signals were converted into corresponding time-frequency diagrams using four different time-frequency analysis techniques. Finally, the time-frequency graph data were used as the input of the deep residual network ResNet18, and intelligent fault diagnosis of the turbine rotor was carried out based on transfer learning. The main conclusions of this study are as follows:

(1) The proposed FEMATL method can realize intelligent diagnosis with high accuracy for turbine rotor fault detection with insufficient fault samples, eliminate the need for a large amount of training data typically required by deep learning models, and solve the problem of small samples in the intelligent diagnosis of turbine rotors.

(2) In this study, four time-frequency analysis techniques, the short-time Fourier transform, continuous wavelet transform, generalized S-transform, and Wigner–Ville distribution, were used to transform the one-dimensional vibration signal of the rotor-bearing system into the corresponding time-frequency diagrams, and the diagnostic effects of the four different time-frequency diagrams were observed.

(3) A well-built finite element model enables the calculation and simulation of a large number of fault samples, even for signals that are difficult or impossible to obtain through physical measurements. Finite element simulations can be very flexible for producing fault samples and expanding fault types. In contrast, transfer learning requires only a small number of fault samples, which can help the model shorten the training time and improve diagnostic accuracy.

In summary, the FEMATL method proposed in this paper can help mechanical devices with insufficient fault samples, such as turbine rotors, to achieve small-sample fault diagnosis with a short training time and high diagnostic accuracy.

## Figures and Tables

**Table 1:** Material parameters of the rotor.

Material Parameters	Shaft	Disc
Density of material	7850 kg/m^3^	7850 kg/m^3^
Modulus of elasticity	2.05 × 10^11^ Pa	2.05 × 10^11^ Pa
Poisson’s ratio	0.3	0.3
Mass	25.93 kg	33.78 kg

**Table 2:** Rotor-bearing system components and corresponding element types.

Rotor System Components	Type
Shaft	Beam189
Disc	Mass21
Sliding bearing	Combi214

**Table 3:** Samples of ten different faults and their labels.

Fault Type	Label	Fault Type	Label
1.0 mm_parallel misalignment	0	90 N_rubbing	5
1.5 mm_parallel misalignment	1	0.1 kg_unbalance	6
2.0 mm_parallel misalignment	2	0.2 kg_unbalance	7
50 N_rubbing	3	0.3 kg_unbalance	8
70 N_rubbing	4	health	9

**Table 4:** Rotor-bearing system failure dataset descriptions.

Dataset	STFT Dataset	CWT Dataset	GST Dataset	WVD Dataset
fault category	10	10	10	10
TFD dimension	875 × 656 × 3	875 × 656 × 3	875 × 656 × 3	875 × 656 × 3
number of training set samples	3000	3000	3000	3000
number of samples in the validation set	1000	1000	1000	1000
number of samples in the test set	1000	1000	1000	1000

**Table 5:** Validation set accuracy in each epoch.

Epochs	1	2	3	4	5	6	7	8	9	10
STFT	0.228	0.772	0.854	0.884	0.973	0.954	0.995	0.998	0.998	0.997
CWT	0.432	0.798	0.905	0.974	0.968	0.977	0.973	0.997	0.995	0.998
GST	0.424	0.832	0.915	0.948	0.996	0.982	0.987	0.985	0.997	0.998
WVD	0.229	0.722	0.722	0.915	0.964	0.963	0.957	0.929	0.985	0.977

**Table 6:** Evaluation indexes of different fault categories in CWT TFD dataset.

Fault Type	Precision	Recall	Specificity	F1
0	1.0	0.99	1.0	0.995
1	1.0	1.0	1.0	1.0
2	1.0	1.0	1.0	1.0
3	1.0	1.0	1.0	1.0
4	1.0	1.0	1.0	1.0
5	1.0	1.0	1.0	1.0
6	0.99	1.0	0.999	0.995
7	1.0	1.0	1.0	1.0
8	1.0	1.0	1.0	1.0
9	0.99	0.99	0.999	0.99

**Table 7:** The impact of transfer learning on rotor fault diagnosis.

Dataset		Epochs	Validation Set Accuracy	Training Time	Test Time
Fault diagnosis using transfer learning	STFT dataset	10	99.8%	399.73/s	10.68/s
	CWT dataset	10	99.8%	397.12/s	10.54/s
	GST dataset	10	99.8%	401.26/s	10.77/s
	WVD dataset	10	98.5%	412.35/s	11.03/s
Fault diagnosis without using transfer learning	STFT dataset	100	87.5%	63.76/min	11.23/s
	CWT dataset	100	88.2%	64.47/min	10.66/s
	GST dataset	100	87.1%	64.52/min	10.89/s
	WVD dataset	100	82.3%	65.01/min	11.78/s

## Data Availability

Not applicable.
